# The local and systemic cytokine signatures of juvenile idiopathic arthritis are attributable to tcr-independent activation of two novel subsets of prematurely senescent t cells found in synovial fluid

**DOI:** 10.1186/1546-0096-12-S1-P35

**Published:** 2014-09-17

**Authors:** Abbe Vallejo, Ian Ferguson, Patricia Griffin, Jeffrey Dvergsten, Joshua Michel, Margalit Rosenkranz, Daniel Kietz

**Affiliations:** 1 Pediatrics, University of Pittsburgh School of Medicine, Pittsburgh, USA; 2 Pediatrics, Duke University, Durham, USA

## Introduction

Cytokine upregulation is considered a hallmark of the autoimmune and inflammatory manifestations in Juvenile Idiopathic Arthritis (JIA). Whilst T cells are thought contribute to cytokine dyscrasia, the underlying mechanism(s) is poorly understood.

## Objectives

We have reported recently that JIA patients carry high numbers of unusual senescent CD8 T cells bearing CD31, a molecule known mediate leukocyte diapedesis into sites of injury. In the present work, we re-surveyed the T cell populations of patients for other CD31^+^ senescent T cell subsets. We hypothesized that CD31 signaling in these senescent cells is a self-perpetuating mechanism for the upregulation of inflammatory cytokines in JIA.

## Methods

Blood and/or synovial fluid (SF) were collected from children with oligoarticular (Oligo) and polyarticular (Poly) JIA. Blood was also collected from age-matched healthy children. By multiplex analysis, cytokine profiles of plasma and SF were determined. By multicolor flow cytometry, abT cell phenotypes in blood and SF were examined. Based on results of the multiplex assay, receptor crosslinking bioassays for cytokine production, and proteomic screening for signaling substrates were performed using primary SF T cells, and transformed T cells expressing CD31.

## Results

The cytokine signature of JIA is characterized by the dominance of IL-6, IL-10 and TNFa in both blood and SF. In addition to CD28^-^CD31^+^ CD8 abT cells, we found a novel subset of DN abT cells that were CD4^-^CD8^-^CD28^-^, but were CD31^+^. CD31 receptor cross-linking of fresh SF T cells enriched for these ab T cell subsets showed specific induction of IL-6, IL-10, IL-17, TNFa, and IFNg. These responses were accompanied by phosphorylation of several signaling substrates including ZAP70 and RelA. Specificity of TCRab-independent, CD31-driven activation was verified by similar bioassays using somatic T cell line mutants expressing CD31, but deficient in TCRab or CD3. Pharmacologic inhibitors of signaling substrates abrogate protein phosphorylation as well as cytokine production, indicating that CD31 ligation sufficiently and effectively bypass conventional TCR-mediated route of T cell activation.

## Conclusion

Blood and SF cytokine profile of JIA is dominated by five cytokines. Such profiles are recapitulated by CD31-driven activation of senescent CD8 and DN abT cell subsets. TCR-independent CD31-driven cellular activation indicates maladaptive T cell function in JIA. Further investigation on the CD31 signaling cascade may pave way to innovations in cell-targeted therapy in JIA.

[This study is supported by grants from the Nancy E. Taylor Foundation for Chronic Diseases, and the US National Institutes of Health].

## Disclosure of interest

None declared.

